# Effect of crystallization temperature and holding time on flexural strength, color and translucency of nano lithium disilicate glass ceramic

**DOI:** 10.1186/s12903-025-06518-w

**Published:** 2025-07-11

**Authors:** Nouran Gamal, Ingy Nouh, Mohamed Eldemellawy, Amr EL-Etreby

**Affiliations:** 1 Department of Fixed Prosthodontics, Faculty of Dentistry, MSA University, Cairo, Egypt; 2 Department of Fixed Prosthodontics, Faculty of Dentistry, Ain Shams University, Cairo, Egypt

**Keywords:** Amber mill, Crystallization temperature, Color change, Microstructure, Flexural strength, Holding time

## Abstract

**Background:**

Ceramists often deviate from the standard crystallization temperatures and holding times recommended by manufacturers, but the effects of these variations remain unclear. The aim of this in-vitro study was to evaluate the effect of different crystallization temperatures and holding times on the color reproduction, translucency and biaxial flexural strength (BFS) of novel lithium disilicate glass ceramic (Amber Mill). In addition, microstructural changes resulting from these altered firing conditions were analyzed using X-ray diffraction (XRD) and scanning electron microscopy (SEM).

**Methods:**

Fifty-six Amber Mill ceramic discs (*n* = 56) of 12 mm diameter and 1 mm thickness in shade A2 were prepared and divided into two main groups according to the final crystallization temperature (*n* = 28); Group (CT1): the manufacturer recommended crystallization temperature 825 °C and Group (CT2): the suggested crystallization temperature 800 °C. Each group was further subdivided into four subgroups according to the holding time (*n* = 7); subgroup (H1): 15 min (as recommended by the manufacturer) and other suggested holding times, subgroup (H2): 20 min, subgroup (H3): 25 min and subgroup (H4): 30 min. Color reproduction and translucency were measured using a spectrophotometer. Color differences (ΔE_00_) and relative translucency parameter (RTP) values were calculated. BFS was measured using Piston-on-three ball test. Crystalline structure and microstructural features present in the material were described using XRD and SEM. Two-way ANOVA followed by Tukey’s post hoc test at significance level of *p* < 0.05 was perfomed.

**Results:**

Decreasing crystallization temperature decreased color change and decreased translucency of Amber Mill with no influence on flexural strength. Increasing holding time led to decrease in color change with no effect on translucency and flexural strength of Amber Mill.

**Conclusions:**

The Crystallization temperature suggested in this study showed superior results in terms of color change when compared to the manufacturer’s recommendations, with reduced translucency and no significant impact on the flexural strength of Amber Mill. Suggested holding times decreased color change without affecting either translucency or flexural strength of Amber Mill.

## Background

Lithium disilicate based glass ceramics (LDC) have been widely used in the field of restorative and esthetic dentistry in recent years. Due to their excellent esthetics, high strength, chemical composition and favorable processing properties, LDCs are suitable for a variety of indications, making them very popular among dental practitioners and technicians [1]. LDCs are available in pressable or machinable form that can be milled using computer-aided design and computer-aided manufacturing (CAD-CAM) technology. Machinable LDC blocks are highly desirable as they enable chairside CAD-CAM dentistry, allowing for the delivery of the final restoration in a single appointment [2]. These blocks are partially crystallized during manufacturing, containing lithium metasilicate crystals (Li_2_SiO_3_), which makes the material easier to mill and reduce wear on milling tools. After milling, the material must undergo a crystallization cycle with a specific holding time in a ceramic furnace to convert lithium metasilicate into lithium disilicate (Li_2_Si_2_O_5_), enhancing the material’s flexural strength [3]. The crystallization process involves phase transformation of the amorphous atomic structure of glass to a well-ordered crystalline structure. During firing, nucleation and growth of crystals within the matrix takes place with minimal dimensional changes. Therefore, crystallization temperature must be precisely controlled to improve mechanical strength of the ceramic [4]. Holding time refers to the duration during which the material is exposed to a specific temperature in the furnace, allowing for mass transfer, grain boundary migration, and crystal growth. An appropriate holding time helps eliminate pores and enhance densification. However, excessively long holding time may lead to abnormal grain growth, reducing material density and negatively affecting mechanical properties. Hence, holding time must be carefully optimized [5].

IPS e. max CAD is the most widely recognized LDC on the market, first introduced in 2006. It has a flexural strength 350 MPa and significantly greater fracture resistance 2.11 MPa m ½ compared to earlier adhesive glass ceramics. Since its introduction, the dental market has been constantly evolving with newer LDCs being introduced rapidly [6]. Amber Mill (NLD; HASS Bio, South Korea) is a recently introduced lithium disilicate CAD-CAM block that incorporates a nanotechnology to allow translucency adjustments within a single block by altering crystallization temperatures. This feature provides restorations with a more natural appearance and multichromatic gradations [7, 8]. Amber Mill is characterized by a dense, highly cross-linked crystal structure making them superior in strength when compared to other LDCs. It also exhibits high esthetic quality, structural durability and edge stability with reduced chipping [9]. Its biaxial flexural strength reaches 250 MPa immediately after milling and increases to 450 MPa following final heat treatment, making it suitable for veneers, onlays, anterior and posterior crowns and three unit bridges up to the second premolar [10]. Yin et al. in 2019 [11], studied the mechanical properties of five CAD-CAM dental materials; Amber Mill, Vita Enamic, Katana Avencia, Amber Mill Hybrid and Lava Ultimate. Amber Mill showed the highest BFS values up to 530 MPa, indicating its use in the posterior region with capability to withstand the chewing forces. Esthetics and mechanical properties are critical for the clinical success of ceramic restorations. A primary goal of esthetic dentistry is the lifelike reproduction of natural teeth and the surrounding tissues. The esthetic outcome of a ceramic restoration depends largely on its translucency, optical properties and color stability, all essential for long-term success, as color changes can compromise restoration quality [12]. Spectrophotometers are used to objectively evaluate color by directing light onto a surface and measuring the intensity of the reflected wavelengths [13]. For in-vitro studies, spectrophotometers are preferred due to their higher consistency and accuracy compared to prefabricated shade guides, which are subjected to clinician interpretation and external factors like lighting, eye fatigue and aging [14]. Color difference (ΔE_00_) relative to a reference, allows comparison of color reproduction across materials. The ΔE_00_ formula is preferred over the older ΔE_76_, as it better correlates with human visual perception [15]. Besides optical properties, the mechanical strength of the material is essential. Fracture is one of the most common causes of failure in all-ceramic restorations, making flexural strength a key property for ensuring fracture resistance and long-term function. Flexural strength refers to the maximum stress a material can withstand under a bending load [16]. Biaxial flexural strength test is often preferred over uniaxial tests, as it simulates multiaxial stresses encountered clinically and avoids bias from crack propagation in a single direction [17]. Piston-on-three ball is a widely accepted biaxial method due to its consistent sample-to-loading tip contact and insensitivity to friction between the supporting balls and the specimen [18]. Color, translucency and mechanical properties can be influenced by several factors, including microstructure, glass composition, crystalline content and crystal size [19]. X-ray diffraction (XRD) is widely used to analyze crystal structures and atomic spacing by detecting the constructive interference of monochromatic X-rays with the crystalline components of a sample. These X rays are generated through a cathode ray tube, filtered to produce monochromatic radiation, then collimated to concentrate, and directed toward the specimen [20]. Scanning electron microscopy (SEM), is another valuable tool for microstructural evaluation of organic and inorganic materials at the nano to micrometer (µm) scale. SEM produces high-resolution images (up to 300,000X magnification) by digitally capturing and analyzing surface topography through modern imaging software [21]. Jung et al. in 2021 [22], evaluated the effect of different crystallization temperatures on microstructure, translucency, and flexural strength of lithium disilicate ceramics. They found that different crystallization temperatures affected optical and mechanical properties of ceramics. Higher crystallization temperatures (860 °C) resulted in larger crystal size, lower translucency and reduced flexural strength. In contrast, lower temperatures (815 °C) resulted in smaller crystal size, greater translucency and higher flexural strength. Demirel et al. in 2022 [23], compared the color stability, translucency and biaxial flexural strength of three lithium disilicate based ceramics; CEREC Tessera, IPS e. max CAD and Vita Suprinity after coffee thermocycling. They concluded that all materials exhibited acceptable color changes regardless of coffee thermocycling, with IPS e.max CAD showing the highest translucency and comparable BFS to CEREC Tessera, while Vita suprinity had the highest BFS. Currently, there are limited studies [24, 25, 8] examining the properties of Amber Mill. Therefore, the aim of this in-vitro study was to evaluate the effect of different crystallization temperatures and holding times on the color reproduction, translucency and biaxial flexural strength of Amber Mill. In addition, microstructural changes resulting from these altered firing conditions were analyzed using X-ray diffraction (XRD) and scanning electron microscopy (SEM).

The null hypothesis was that varying crystallization temperatures and holding times would not affect color, translucency or biaxial flexural strength of Amber Mill, nor result in difference in its microstructure or crystalline content.

## Methods

A 14 mm partially crystallized lithium disilicate glass ceramic block (Amber Mill; HASS Bio, South Korea, Batch number; EBE06NF 1601) in A2 shade, chemical composition (SiO_2_: <78%, Li_2_O: <12%, coloring oxides: <12%) was selected for this study. Sample size calculation was conducted using a software program (G*Power version 3.1.9.7) [26]. By adopting an alpha (α) level of (0.05), a beta (β) of (0.05) (i.e. power = 95%), and an effect size (f) of (0.595) calculated based on the results of a previous study [27]; the predicted sample size (n) was found to be (56) samples (i.e. 28 samples per group and 7 samples per subgroup). Fifty-six Amber Mill ceramic discs (*n* = 56) were prepared according to the ISO 6872 specifications for testing ceramic materials [28]. First, ceramic blocks were milled into a cylindrical shape of 12 mm diameter using a tool grinder. Ceramic cylinders were inspected after milling for any surface flaws. Dimensions were confirmed following milling by digital caliper (Model 6784ec, Se, China). Using a slow speed diamond saw (IsoMet 4000, Buehler, USA), each cylinder was sectioned into discs of 1 mm thickness under copious cooling. Ceramic specimens were inspected after sectioning for any surface flaws. Dimensions and thickness were confirmed after sectioning using digital caliper [23, 29]. All the specimens were prepared by the same operator for the purpose of standardization. Specimens were divided into two main groups according to the final crystallization temperature CT/ ^°^C (*n* = 28); Group (CT1): the manufacturer recommended crystallization temperature 825 °C and Group (CT2): the suggested crystallization temperature 800 °C. Each group was further subdivided into four subgroups according to the holding time H /min (*n* = 7); subgroup (H1): 15 min (as recommended by the manufacturer) and other suggested holding times, subgroup (H2): 20 min, subgroup (H3): 25 min and subgroup (H4): 30 min. All heat treatments were performed using a Programat CS 3 ceramic furnace (Ivoclar Vivadent, Schaan, Liechtensetin) following the manufacturer recommended firing cycle; Standby temperature (B): 400 °C for 3 min, the heating rate: 60 °C /min, cooling rate: 690 °C and vaccum; V1: 550 °C and V2: 825 °C and were remain fixed in all groups. Two different crystallization temperatures with four different holding times were tested as follows; CT1H1: 825 °C and 15 min, CT1H2: 825 °C and 20 min, CT1H3: 825 °C and 25 min, CT1H4: 825 °C and 30 min, CT2H1: 800 °C and 15 min, CT2H2: 800 °C and 20 min, CT2H3: 800 °C and 25 min and CT2H4: 800 °C and 30 min. After completion of firing, specimens were removed from the furnace and allowed to cool to room temperature.

### Color evaluation

A digital spectrophotometer (VITA Easyshade V, VITA Zahnfabrik, Bad Säckingen, Germany) was used to measure each specimen’s CIELAB coordinates while they were placed over a neutral gray (L*: 51.73, a*: 1.01, and b*: 3.14) background under standardized ambient lighting (6500 K daylight-simulating fluorescent light source). Three measurements were taken for each specimen at the center and their average was recorded. Before each measurement, the spectrophotometer was recalibrated according to the manufacturer’s recommendation. All measurements were made by the same operator. Color difference (ΔE_00_) was determined after comparing the standard Vita coordinates (L*: 61.28, a*: 0.92, and b*: 9.27) of the A2 shade [30] stored in the Easyshade [31].

### Translucency evaluation

A quantitative measurement of translucency was obtained by measuring the CIELAB coordinates of the specimens over a white (L*: 91.90, a*: 1.42, and b*: 8.29) and black (L*: 8.52, a*: 0.32, and b*: 0.65) background using Vita Easyshade V spectrophotometer (VITA Zahnfabrik, Bad Säckingen, Germany). For each specimen, three measurements were taken and their average was recorded. CIEDE2000 formula was used to calculate translucency parameter (RTP):

CIEDE2000 = [(Δ*L*′ ∕ *k*_L_*S*_L_) ^2^ + (Δ*C*′∕ *k*_C_*S*_C_) ^2^ + (Δ*H*′∕ *k*_H_*S*_H_) ^2^ + *R*_T_ (Δ*C*′ ∕ *k*_C_*S*_C_) (Δ*H*′∕ *k*_H_*S*_H_) ]^1 ∕ 2^.

Where ΔL′, ΔC′, and ΔH′ are the differences in the lightness, chroma, and hue of a specimen. R_T_ refers to the interaction between the chroma and hue differences in the blue region. Weighting functions of S_L_, S_C_, and S_H_ are used to adjust the total color difference. The parametric factors (k_L_, k_C_, and k_H_) were set as 1 [23].

### Biaxial flexural strength test

A universal testing machine (Instron 3345, Norwood, USA) was used to test specimens for BFS using the piston-on-three-ball technique. Specimens were supported by a custom-made metallic platform of 10 mm diameter with three 3.2 mm spherical stainless-steel balls, spaced 120^°^ apart. Following ISO 6872, a piston with 1.6 mm round end, applied load to the specimen’s center at a crosshead speed of 1 mm/min [28]. A thin plastic polyethylene sheet was positioned to promote uniform load distribution and reduce surface contact damage between the supporting balls and the specimen as well as between the loading piston and the specimen. Load was continuously applied until the ceramic fractured and the fracture load for each specimen was recorded. BFS was calculated using the following equation [27]:

S= [-0.2387P(X-Y)]/d^2^.

Where S; maximum tensile stress (MPa), P; fracture load (N), d; specimen disk thickness at fracture origin (mm). X and Y were determined as follows:

X = (1 + v) In (r_2_ ∕ r_3_) ^2^ + [(1 − v) ∕ 2] (r_2_ ∕ r_3_) ^2^.

Y = (1 + v) [1 + In (r_1_ ∕ r_3_) ^2^] + (1 − v) (r_1_ ∕ r_3_) ^2^.

Where v is the Poisson’s ratio, r_1_ is the radius of the support circle (mm), r_2_ is the radius of the tip of the piston (mm), and r_3_ is the radius of the specimen (mm). Poisson’s ratio for tested materials, which is 0.24 for lithium disilicate ceramic material was taken from a previous report.

### X-ray diffraction

XRD using a diffractometer was used to evaluate the type of crystals found in the material and their amount [32]. Specimens of each group were placed on the holder of the diffractometer (X pert pro, USA; PW 3040/60) and scanned utilizing Cu Kα x-ray angle from 20 to 40 degrees, 2θ with a step size of 0.04 degrees and 5s-step interval. Crystallized volume fraction (CF - %) was calculated from the ratio of the crystalline area (A_C_) to the total area (A_T_) of the diffractograms using the Equation below [33]:

CF= (A_C_ /A_T_) x 100.

### Microstructure by SEM

For microstructural analysis, specimens of each group were subjected to scanning electron microscope (Quattro SEM, Thermo Fisher Scientific, USA). Specimens were polished using a white disc to remove any surface debris, etched with 9.5% Hydrofluoric acid (Porcelain etchant, Bisco, USA) for 20 s, rinsed with air water spray for 1 min, air dried, to enhance glassy matrix dissolution with higher exposure of lithium disilicate crystals, cleaned in ultrasonic cleaner for 3 min for enhancing image clarity [1], then sputter coated with gold-palladium (Au-pd) (DST1-170, Vac Techniche, UK) at 700 w, 220 V and 50 Hz for enhancing sample surface conductivity, ensuring accurate imaging, and better resolution during electron microscopy [33]. Images of the specimens were obtained using SEM at an accelerating voltage of 30 kV to assess grains at magnification of 40,000X.

### Statistical analysis

Numerical data were presented as mean and standard deviation (SD) values. They were explored for normality and variance homogeneity by checking the data distribution and using Shapiro-Wilk’s and Levene’s tests, respectively. Biaxial flexural strength data were not normally distributed and were transformed using Box-cox transformation to achieve normality. Other data were normally distributed with homogenous variances. Data were analyzed using two-way ANOVA followed by Tukey’s post hoc test. Simple effects comparisons were made utilizing the ANOVA error term with p-values adjustment using the False Discovery Rate (FDR) method. The significance level was set at *p* < 0.05. Statistical analysis was performed with R statistical analysis software version 4.4.0 for Windows [34].

## Results

Descriptive data for different measured outcomes are presented in (Table 1).

**Table 1:** Descriptive statistics

Measurement	Crystallization temperature	Holding time	Mean	95% confidence intervals		SD
				Lower	Upper	
Color change	CT1	H1	10.09	9.94	10.23	0.19
		H2	9.40	9.25	9.54	0.20
		H3	9.02	8.87	9.17	0.21
		H4	8.83	8.66	8.99	0.22
	CT2	H1	7.79	7.64	7.94	0.20
		H2	8.50	8.35	8.66	0.21
		H3	8.04	7.90	8.19	0.19
		H4	7.79	7.64	7.94	0.20
Translucency	CT1	H1	10.56	10.45	10.67	0.15
		H2	10.59	10.48	10.70	0.15
		H3	10.58	10.49	10.67	0.12
		H4	10.60	10.50	10.71	0.15
	CT2	H1	10.32	10.23	10.40	0.12
		H2	10.31	10.20	10.43	0.16
		H3	10.39	10.28	10.49	0.14
		H4	10.34	10.21	10.46	0.16
Biaxial flexural strength	CT1	H1	1098.91	1002.54	1195.28	130.09
		H2	1277.66	989.93	1565.39	388.41
		H3	1170.08	1046.49	1293.68	166.84
		H4	1082.38	1010.15	1154.60	97.49
	CT2	H1	1045.31	965.43	1125.20	107.83
		H2	978.97	846.89	1111.05	178.29
		H3	1066.39	981.78	1150.99	114.20
		H4	1163.01	1115.57	1210.44	64.03

Two-way ANOVA tests revealed that for color change and flexural strength, there was a significant interaction between crystallization temperature and holding times (*p* < 0.001 and 0.041, respectively). However, for translucency, only the main effect of the temperature was statistically significant (*p* < 0.001) (Table 2).

**Table 2:** ANOVA tests

Measurement	Source	Sum of squares	df	Mean square	f-value	*p*-value
Color change	Crystallization temperature	23.78	1	23.78	**579.13**	< 0.001*
	Holding time	4.19	3	1.40	**34.05**	< 0.001*
	Crystallization temperature * holding time	4.67	3	1.56	**37.91**	< 0.001*
Translucency	Crystallization temperature	0.84	1	0.84	**40.55**	< 0.001*
	Holding time	0.02	3	0.01	**0.28**	**0.838**
	Crystallization temperature * holding time	0.01	3	0.00	**0.22**	**0.879**
Biaxial flexural strength	Crystallization temperature	4.38	1	4.38	**3.98**	**0.052**
	Holding time	1.55	3	0.52	**0.47**	**0.704**
	Crystallization temperature * holding time	9.81	3	3.27	**2.98**	0.041*

df degree of freedom, * Significant (*p* < 0.05)

### Color change (ΔE_00_)

**Fig. 1:**
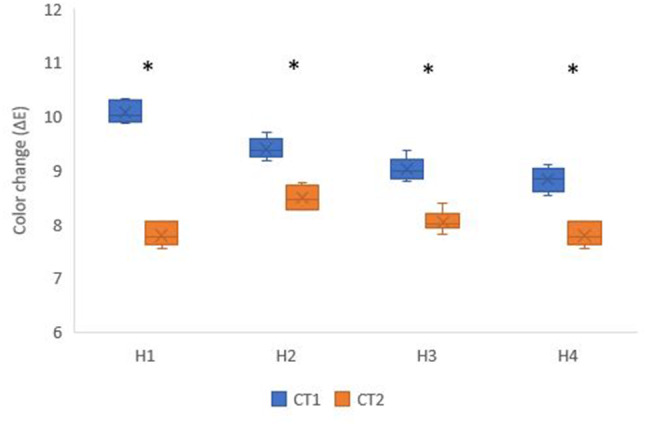
Box plot showing color change (ΔE_00_) values for different crystallization temperatures and holding times; * Statistically significant difference between crystallization temperatures

CT1 (9.33 ± 0.53) had a significantly higher color change (ΔE_00_) than CT2 (8.03 ± 0.35) at *p* < 0.001. Additionally, there was a significant difference between different holding times (*p* < 0.001). The highest color change was found in H2 (8.95 ± 0.50), followed by H1 (8.94 ± 1.21), then H3 (8.53 ± 0.54), while the lowest color change was found in H4 (8.31 ± 0.58). Post hoc pairwise comparisons showed H1 and H2 to have significantly higher color changes than other holding times (*p* < 0.001). In addition, they showed H3 to have significantly higher color change than H4 (*p* < 0.001) (Fig. 1).

### Translucency

**Fig. 2:**
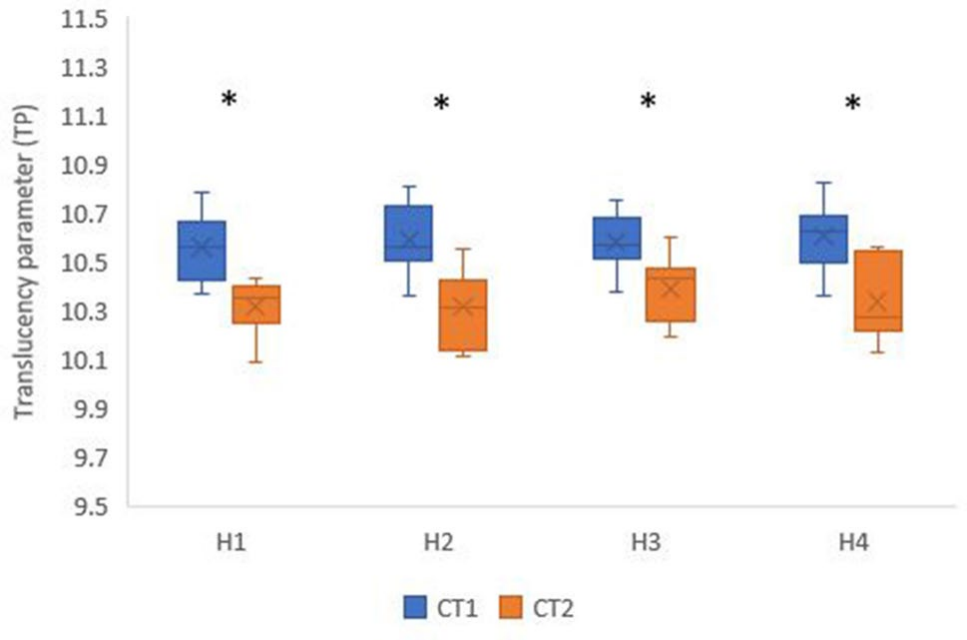
Box plot showing translucency parameter (TP) values for different crystallization temperatures and holding times; * Statistically significant difference between crystallization temperatures

CT1 (10.58 ± 0.13) had a significantly higher translucency than CT2 (10.34 ± 0.14) at *p* < 0.001. While, there was no significant difference between different holding times (*p* = 0.838). The highest translucency was found in H3 (10.48 ± 0.16), followed by H4 (10.47 ± 0.20), then H2 (10.45 ± 0.20), while the lowest translucency was found in H1 (10.44 ± 0.18) (Fig. 2).

### Biaxial flexural strength

**Fig. 3:**
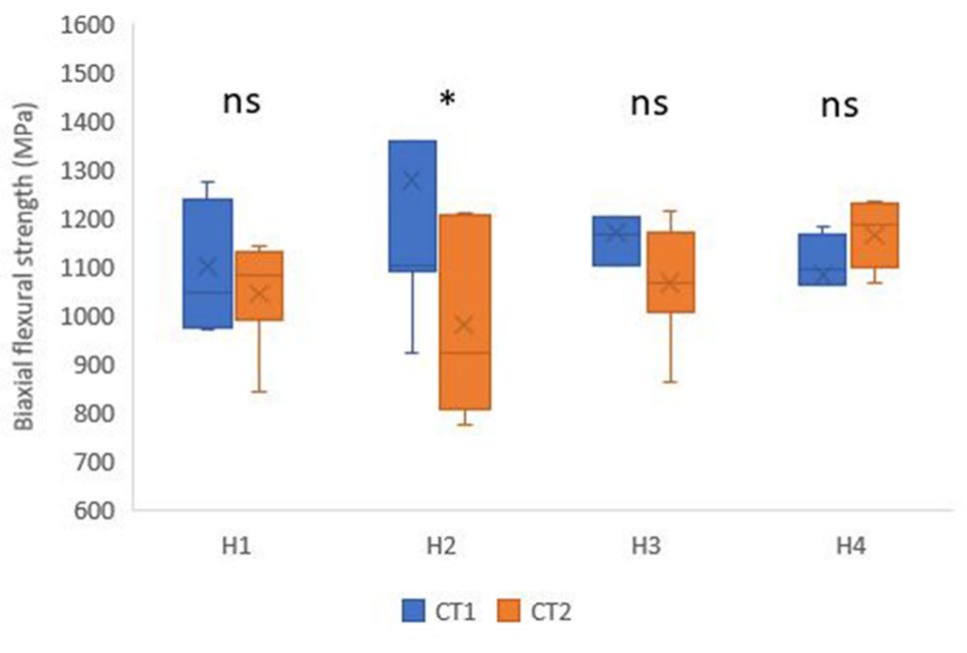
Box plot showing biaxial flexural strength (MPa) values for different crystallization temperatures and holding times; * Statistically significant difference between crystallization temperatures; ns: not statistically significant

CT1 (1108.38 ± 227.42 MPa) had a higher strength value than CT2 (1063.42 ± 134.03), yet the difference was not statistically significant (*p* = 0.052). Additionally, there was no significant difference between different holding times (*p* = 0.704). The highest strength was found in H2 (1128.31 ± 329.12 MPa), followed by H4 (1122.69 ± 89.61 MPa), then H3 (1118.23 ± 147.52 MPa), while the lowest strength was found in H1 (1072.11 ± 118.11 MPa) (Fig. 3).

### X-ray diffraction analysis (XRD)

**Fig. 4:**
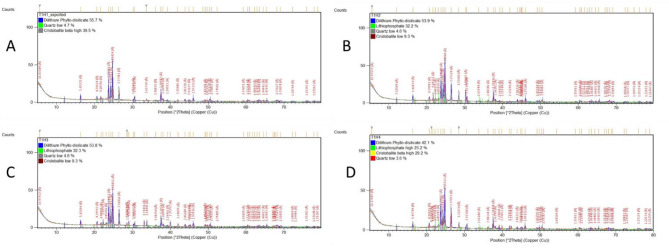
XRD patterns and diffraction intensity of Amber Mill specimens after the manufacturer recommended heat treatment; CT1(825 °C) with different holding times: (**A**); H1:15 min, (**B**); H2: 20 min, (**C**); H3:25 min, (**D**); H4: 30 min

**Fig. 5:**
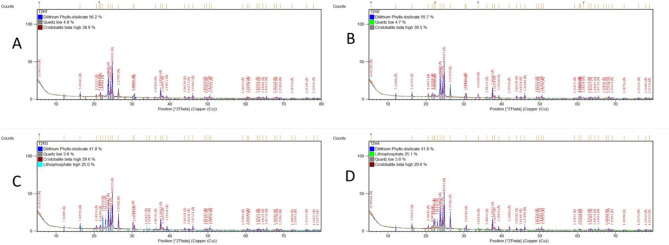
XRD patterns and diffraction intensity of Amber Mill specimens after the suggested heat treatment; CT2 (800 °C) with different holding times: (**A**); H1:15 min, (**B**); H2: 20 min, (**C**); H3:25 min, (**D**); H4: 30 min

XRD examines crystalline material structure, atomic arrangement, crystal size, and imperfections. XRD of all specimens revealed diffraction peaks that correspond to crystalline phases present showing that the material is a predominantly crystalline structure; Dilithium phyllo-disilicate (Li_2_Si_2_O_5_) was found to be the main crystalline phase in all groups. Lithiophosphate, Quartz and Cristobalite crystalline phases were found in groups with varying amounts (Figs. 4 and 5).

### Microstructure by SEM

**Fig. 6:**
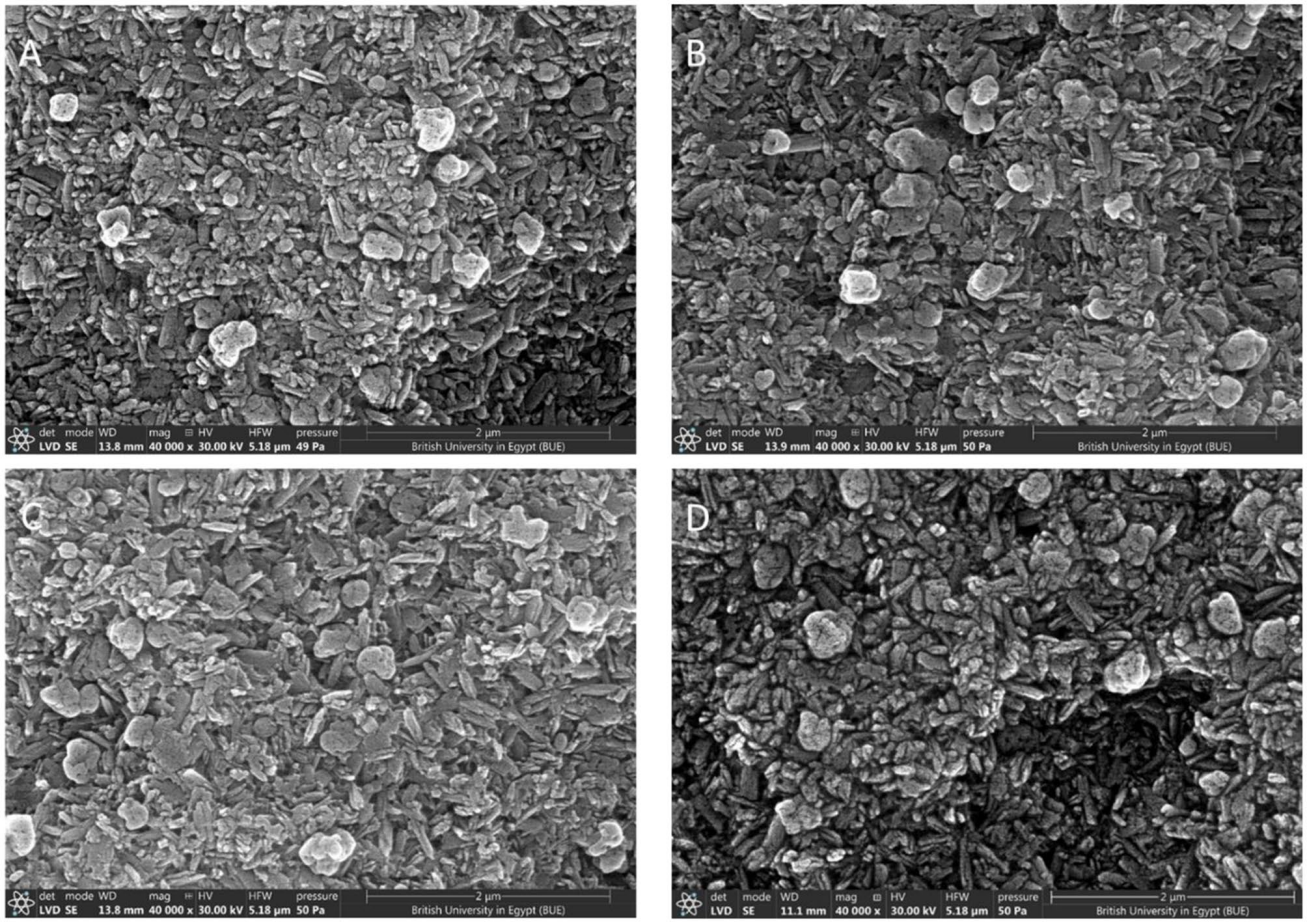
SEM micrographs of Amber Mill specimens after the manufacturer recommended heat treatment; CT1(825 °C) with different holding times: (**A**); H1: 15 min, (**B**); H2: 20 min, (**C**); H3: 25 min, (**D**); H4: 30 min (Magnification 40,000X)

**Fig. 7:**
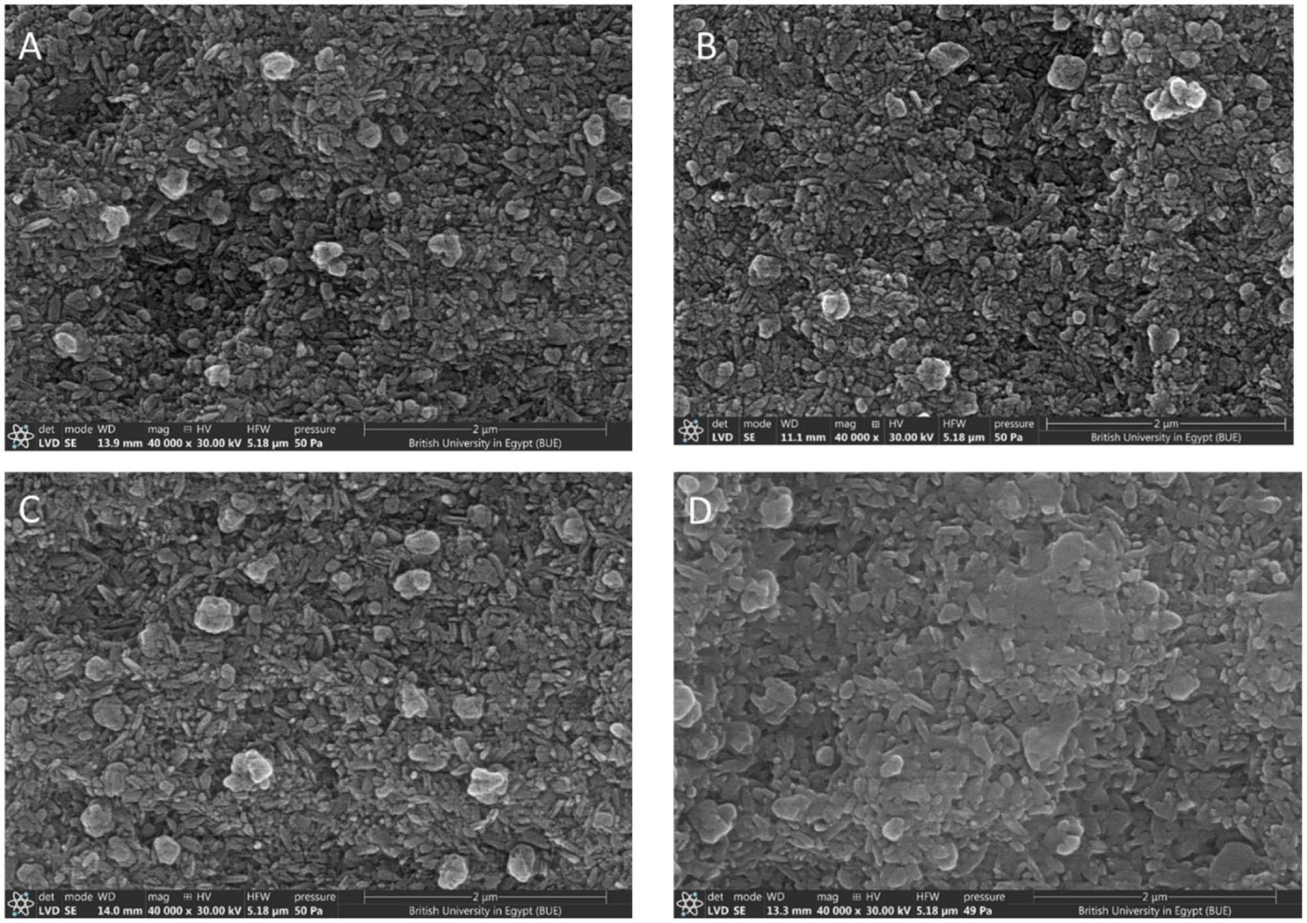
SEM micrographs of Amber Mill specimens after the suggested heat treatment; CT2 (800 °C) with different holding times: (**A**); H1: 15 min, (**B**); H2: 20 min, (**C**); H3: 25 min, (**D**); H4: 30 min (Magnification 40,000X)

Scanning electron microscopic observations of Amber Mill specimens under 40,000X magnification showed crystals of various sizes. In CT1H1 specimens the length of Dilithium phyllo-disilicate crystals averaged (0.308 μm), CT1H2 (0.294 μm), CT1H3 (0.313 μm), CT1H4 (0.323 μm) (Fig. 6), compared to CT2H1 specimens that showed Dilithium phyllo-disilicate crystals averaged (0.210 μm), CT2H2 (0.178 μm), CT2H3 (0.193 μm) and CT2H4 (0.223 μm) (Fig. 7). This shows that there was a noted increase in crystal length from CT2 to CT1.

## Discussion

In this in-vitro study, two crystallization temperatures and four different holding times were used to evaluate their impact on color reproduction, translucency and biaxial flexural strength of Amber Mill. In addition, XRD and SEM were used to identify microstructural difference. According to the results of this in-vitro study, crystallization temperature and holding times had a significant effect on color change and translucency. However, they had no significant effect on biaxial flexural strength with difference in microstructure and crystalline content. Thus, the null hypothesis was partially rejected. Changing the crystallization temperature to 800 °C showed significant lower color change than the crystallization temperature recommended by the manufacturer. As shown by SEM, the lithium disilicate crystals in specimens subjected to the suggested crystallization temperature have an average length less than specimens subjected to the manufacturer crystallization temperature. This might lead to more uniform light distribution and transmission with less or slight color shift. This explanation comes according to a previous study by Jurado et al. in 2022 [13], who reported that the results of SEM observations of Amber Mill ceramic groups suggested that the increase in color shift may be related to the bigger crystals subjected to high firing temperature which in turn induce light scattering causing more color alteration. Also changing the holding time affected the color change, with holding time (H4) showing the most significant lower color change when compared to the other tested subgroups. This can be explained that with further increase in holding time, the crystal size did not increase thus, the color change in H4 group was the least. Increasing the holding times increased the color change, as crystal growth might increase the refractive index mismatch between the glass matrix and the crystalline phase. These results coincide with Fan et al. in 2022 [35]. They stated that increasing holding time led to higher molecular mobility, low glass viscosity, and improved lithium disilicate crystals dislocations. In this way, small crystal agglomerate forming larger crystal sizes. Changing the crystallization temperature significantly affected translucency. Decreasing the crystallization temperature, decreased the translucency of the specimens. This can be attributed to the lesser the crystallization temperature, the smaller the crystal size and the lower the translucency. These results come in accordance with Demirel et al. in 2023 [23], who reported that the crystalline structure has a great influence on translucency since decreasing crystallization temperature led to less crystal size and lower translucency of lithium disilicate ceramics. However, these results contradict with studies such as Miranda et al. in 2020 [14], who stated that crystallization temperature had no influence on translucency and jung et al. in 2021 [22], who reported that lithium disilicate ceramics specimens treated under higher heat temperature showed larger crystal size and lowest translucency. While, specimens treated under lower heat temperature showed smaller crystal size and highest translucency. However, changing the holding time didn’t have a significant effect on translucency of both tested groups. This contradicts with Sun et al. in 2021 [36], who reported that lithium disilicate crystals grow at longer holding time thus increasing the translucency. Changing the crystallization temperature and holding time had no significant effect on flexural strength of tested groups. Therefore, the change in crystal size when changing crystallization temperature and holding time wasn’t significant enough to affect the flexural strength of tested specimens. This might be due to that firing temperature variations were as low as 25 °C, which is insufficient to significantly impact flexural strength through the interlocking effect of the crystalline phase. Additionally, manufacturers incorporate various oxides that act as co-nucleating agents to alter the shade and translucency of glass ceramics. These oxides interact with the nucleation and crystallization processes, thus affecting optical properties without significant effect on mechanical properties when applying minimum change in firing temperatures [37]. These results come in accordance with Akl et al. in 2025 [38], and Abdel Sadek et al. in 2022 [9], who stated that altering crystallization temperatures of the same shade to obtain different translucencies had no significant effect on the flexural strength of Amber Mill CAD. However, these results don’t coincide with Wang et al. in 2019 [39]. They reported that increasing the crystallization temperatures led to increase in the crystal size of lithium disilicate restorative material thus improving flexural strength. Larger crystal size could enhance the interlocking effect of the crystalline phase, which might be beneficial to crack propagation resistance in lithium disilicate ceramics.

### Clinical implications

Optical properties showed superior results when changing crystallization temperature and holding times in comparison to the manufacturer’s recommendations, while significant impact on flexural strength is negligible. The alterations in color and translucency could be beneficial to restorative dentists as desired esthetic outcomes may be achieved without compromising the flexural strength of Amber Mill CAD-CAM restorations.

### Limitations of the study

Limitations of the present in-Vitro study:

- In vitro design that might not properly represent the clinical circumstances. Therefore, in vivo studies are required to reach a definite conclusion regarding the durability of the tested material.
- Constant thickness of the specimens used during the study.

## Conclusions

Within the limitations of this in vitro study, it could be concluded that:

The Crystallization temperature suggested in this study showed superior results in terms of color change when compared to the manufacturer’s recommendations, with reduced translucency and no significant impact on the flexural strength of Amber Mill. Suggested holding times decreased color change without affecting either translucency or flexural strength of Amber Mill.

## Data Availability

All relevant datasets and their supporting information files generated and analyzed during this study are available from the corresponding author upon reasonable request.

## Abbreviations

BFS: Biaxial Flexural Strength
XRD: Xray Diffraction
SEM: Scanning Electron Microscope
ΔE: Color Difference
RTP: Relative Translucency Parameter
LDC: Lithium Disilicate Ceramics
CAD-CAM: Computer-Aided Design / Computer-Aided Manufacturing
Li_2_SiO_3_: Lithium Metasilicate Crystals
Li_2_Si_2_O_5_: Lithium Disilicate Crystals
NLD: Nano Lithium Disilicate
MPa: Megapascal
ISO: International Organization for Standardization
